# A Bone Cyst in the Cervical Region of the Vertebral Column: A Report of a Rare Case

**DOI:** 10.7759/cureus.46534

**Published:** 2023-10-05

**Authors:** Kholoud Sandougah

**Affiliations:** 1 Internal Medicine, College of Medicine, Imam Mohammad Ibn Saud Islamic University, Riyadh, SAU

**Keywords:** tumors, benign, pediatrics, cervical spine, aneurysmal bone cysts, abc, palpable mass, neck pain

## Abstract

Of all primary spine tumors, 15% are benign osteolytic lesions known as aneurysmal bone cysts (ABCs). Owing to the involvement of surrounding neurovascular structures and the potential for cervical spine instability, ABCs in the cervical spine represent a relatively uncommon clinical entity with surgical resection that is extremely challenging. This report details a case of an ABC in the cervical spine affecting a 10-year-old child who presented at the Medical Services Center at Imam Muhammad Ibn Saud Islamic University. The patient manifested with neck pain, a history of trauma, limitations in cervical motion, and neurological changes over the course of clinical follow-up. Diagnostic measures included radiography and computed tomography. The child underwent surgery to stabilize the cervical spine and to excise both the tumor and the affected vertebrae. Given the high recurrence rate of previously described lesions, various additional techniques have been utilized in conjunction with surgical resection, such as radiation and embolization. This paper further discusses the patient's progress, the chosen treatment, and the range of available options. More research is needed to develop evidence-based treatment plans for cervical spine ABCs in younger patients.

## Introduction

Benign tumor-like, expansile lytic lesions known as aneurysmal bone cysts (ABCs) predominantly affect the metaphyses of long bones. These cysts feature a distinct pathology characterized by the presence of blood-filled cavities within the lesion [1]. Notably, 60% of individuals with ABCs are under 20 years of age, making them particularly prevalent among children and adolescents. Seventy percent of ABC cases are primary, meaning they have no underlying lesions. Secondary ABCs are often associated with conditions such as giant cell tumors, chondroblastomas, osteoblastomas, and telangiectatic osteosarcomas [2]. ABCs are relatively rare in the vertebral column, accounting for 12 to 30% of all ABC occurrences, and they primarily affect the posterior elements. A scant 2% of cervical spine cases are attributed to ABCs. The symptoms can vary widely, potentially causing anything from pain to neurological issues, primarily affecting long bones and vertebrae. Due to their proximity to neurovascular structures and the inherent instability in the cervical spine, complete excision typically yields good outcomes but can be challenging to execute [3].

ABCs are most commonly located in the posterior elements at thoracic and lumbar levels. Axial discomfort is the most prevalent sign of the tumor, which progresses quickly and aggressively. Although uncommon, neurological involvement may manifest as spinal instability resulting from bone loss or direct myeloradicular compression. On magnetic resonance imaging (MRI), the presence of fluid-filled multilocular cysts and fluid-fluid levels, along with an expansile lesion bordered by a thin bone shell, often termed the "egg-shell" image, are recognizable features [4].

ABCs have been treated using a variety of methods, including non-surgical approaches like selective arterial embolization, intralesional injections, and radiation therapy, as well as surgical methods like partial intralesional excision and bone graft filling [5]. The cervical spine may not always be amenable to supramarginal resection, which has been shown to reduce the recurrence rate of ABCs by up to 30% [6]. The most effective treatment is still surgical removal, which also controls the area to avoid any relapses and prevents spinal abnormalities.

## Case presentation

**Figure 1:**
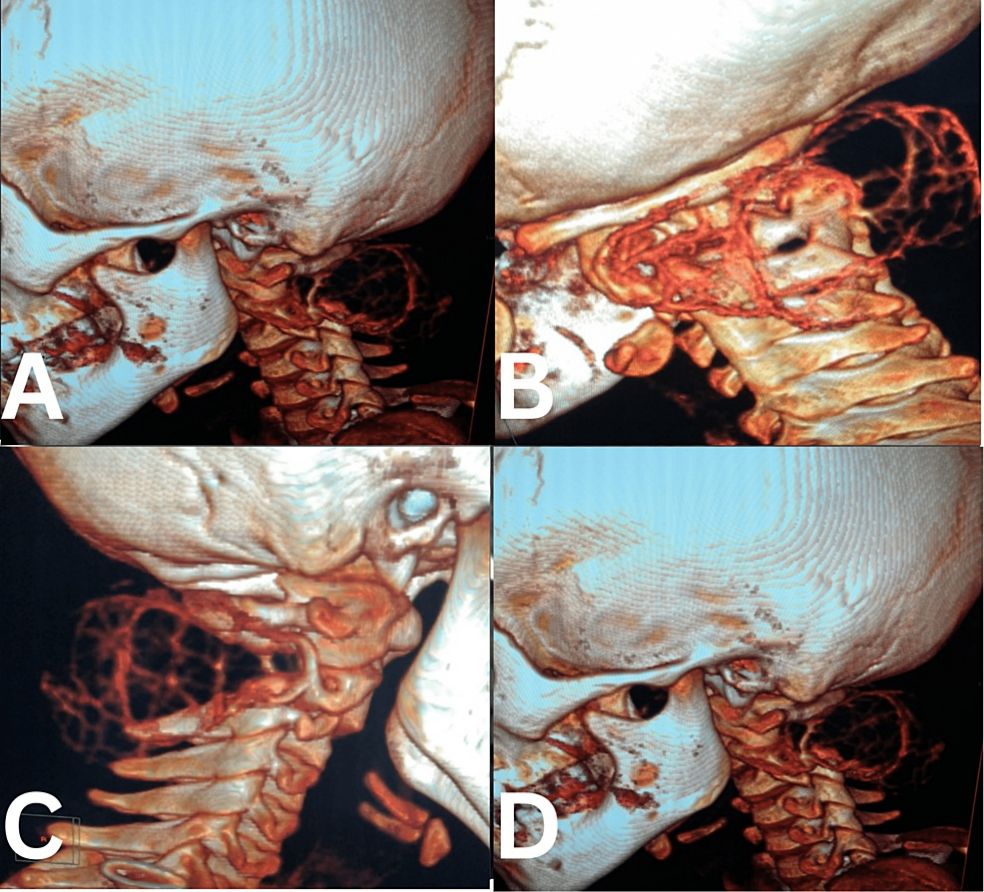
Neck CT scan three-dimensional images. (A, B) sagittal; (C) oblique; (D) coronal sections.

**Figure 2:**
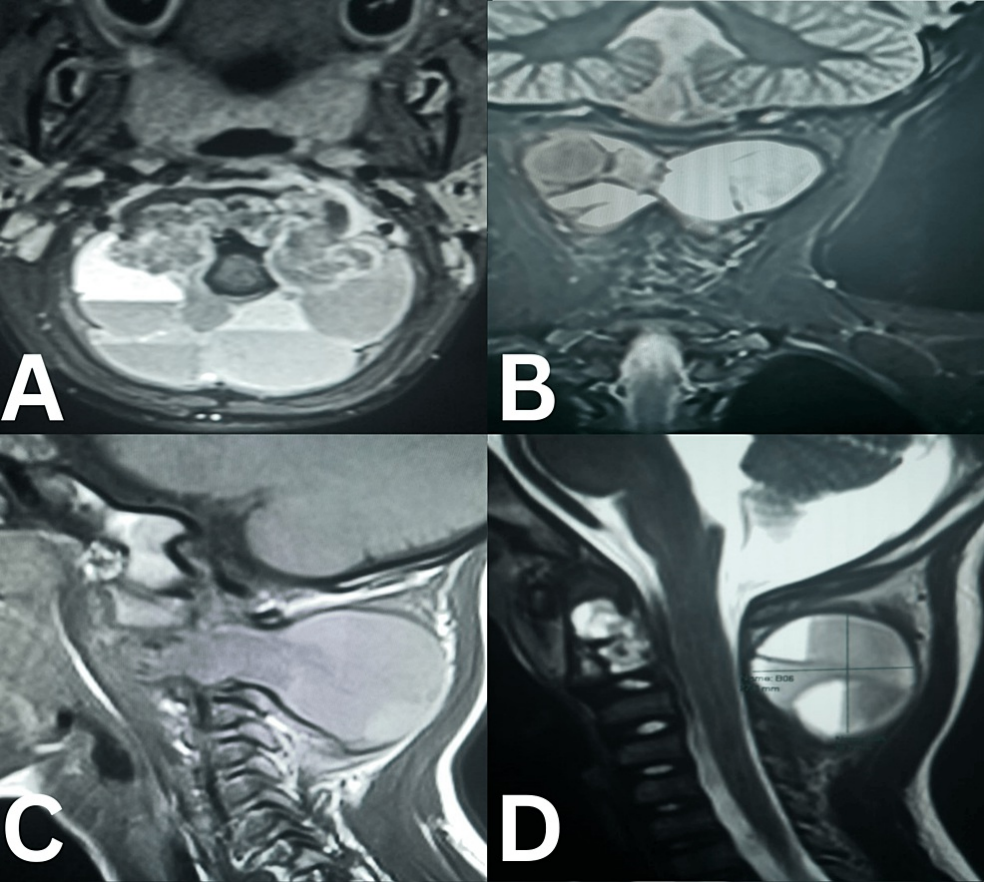
Neck MRI. (A) Axial T2WI demonstrating a large expansile bony lesion with multiple fluid-fluid levels due to blood-filled cavities, separated by septa. (B) T2-W MRI coronal image with variable intensities. (C) T1-W MRI sagittal brain with neck large cyst. (D) T2-W MRI sagittal view with the same finding as in panel C.

**Figure 3:**
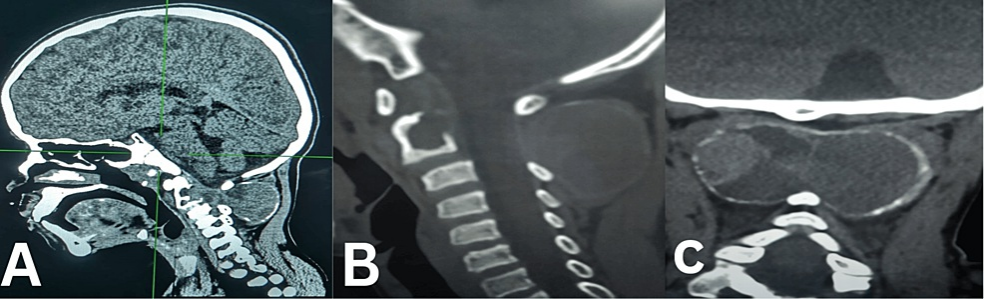
Neck CT scan. (A) Sagittal view with soft tissue window. (B) Sagittal CT bone window with C2 posterior elements, multiloculate bony cyst with osteoclastic changes, and thinned cortices, mainly posterior elements. (C) CT scan coronal view with the same findings as in panel C.

A 10-year-old boy arrived at the Medical Services Center at Imam Muhammad Ibn Saud Islamic University complaining of neck pain, palpable swelling at the nape of the neck, and a history of trauma. The patient described only a restricted range of motion and neck pain that extended to his shoulders as symptoms. No focal neurologic symptoms or signs were present other than neck pain. There were no neurological symptoms such as upper extremity paresthesia or weakness. He was afebrile upon physical examination, and palpation of the posterior cervical region revealed tenderness. Examinations of the cranial nerves and higher mental functions were both normal. Motor and sensory examinations of the lower limbs were also normal, and he exhibited no pathologic reflexes. Biological parameters were within normal limits. According to a local examination, a 6 x 3 cm bony, immovable enlargement was observed in his midline posterior neck, without any localized warmth or discomfort. Neck movement was restricted but without any neurological impairments. A CT scan revealed no fracture, but there was a posterior upper neck dumbbell mass with a C2 osteolytic, septated lesion with calcification. An MRI neck scan also suggested ABC. The spinal cord was unaffected, and bilateral vertebral arteries showed normal flow voids. No signs of spinal canal narrowing were present (Figures 1-3). Given the critical situation, the old hematoma was considered, and the patient was referred for a higher level of care. Open surgical resection was recommended based on the clinical presentation and neuroradiological findings. The decision was made not to immediately perform posterior fusion of the cervical spine, as the location of the lesion primarily implicated the posterior elements in the upper cervical spine, thereby reducing the likelihood of post-surgical instability.

**Figure 4:**
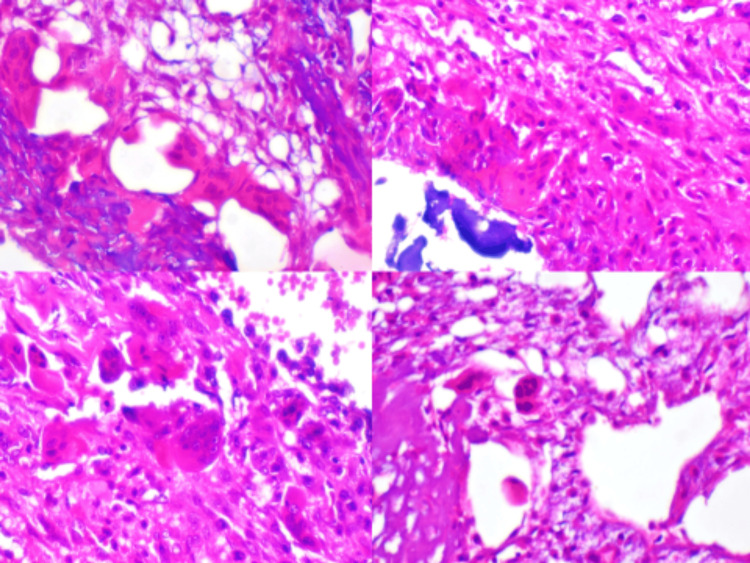
images of histopathologic examination. Different images of histopathologic examination showing characteristic cavernous channels and osteoclastic and osteoblastic reactions.  Additionally, multinucleated giant cells were found surrounding isolated spongious bone fragments that included bone marrow components and largely mature compact bone (hematoxylin and eosin stain x200).

**Figure 5:**
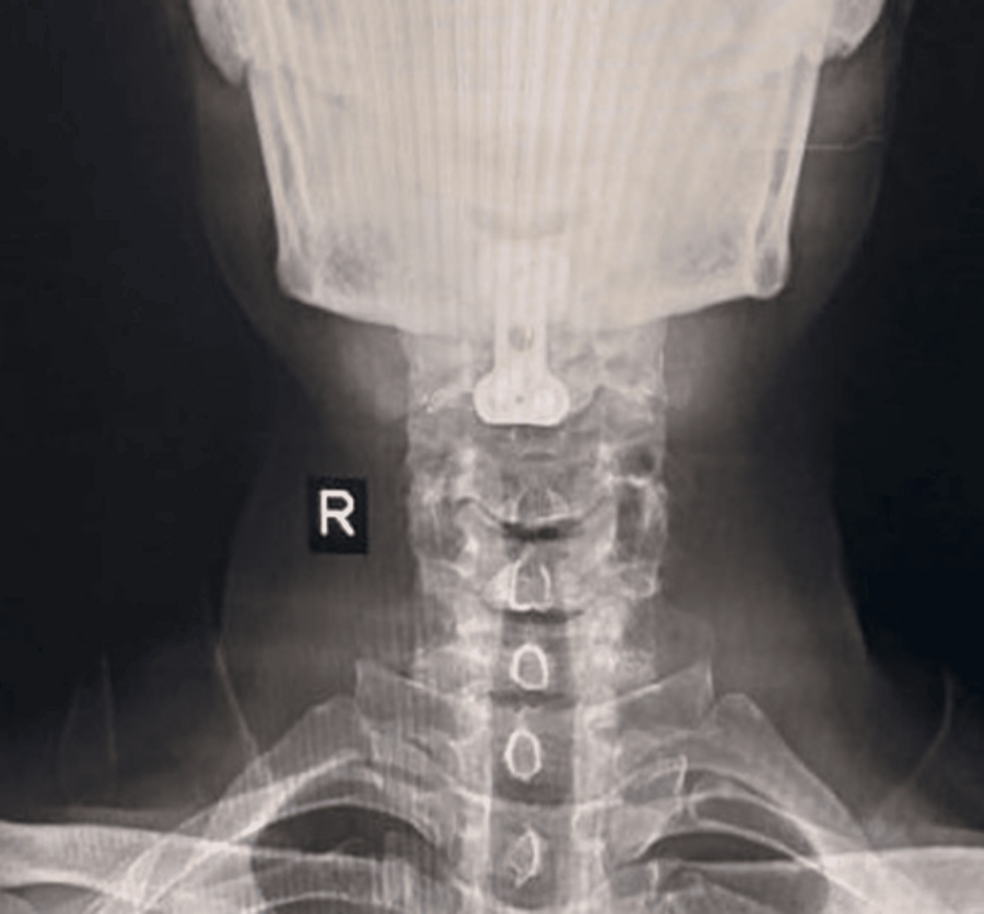
Post-operative X-ray anteroposterior view: one-month follow-up.

A head holder was used to keep the patient in the prone position. A midline skin incision and bilateral subperiosteal muscle dissection exposed an expanding bone lesion, which was then removed along with a C3 laminectomy and adequate decompression of the neural tissues. High-speed drilling was performed on the left facet joint and the afflicted margins of the C2 lamina. There were pockets of varying-stage bleeds in the intratumoral septa of the lesion, but no significant active bleeding occurred during the lesion's removal. After cervical curettage, a bone graft was placed. The defect's stability was ensured by filling it with bone allograft and prescribing a postoperative plastic cervical collar to keep the neck immobilized. Only analgesics were prescribed for the patient. The histological analysis confirmed the presence of an ABC, characterized by cavernous channels and both osteoclastic and osteoblastic responses. The histopathological report noted that mature fat cells, muscle fibers, and connective tissue pieces of tendons with chondroid metaplastic foci were all visible under the microscope. Additionally, localized spongy bone pieces comprising bone marrow components and primarily mature compact bone were observed. The cyst's thin wall was lined with flat epithelial cells that appeared to be mesothelial. No blood cells were found in its hollow. The histological findings supported the diagnosis of an ABC with characteristic cavernous channels and osteoclastic and osteoblastic reactions (Figure 4). One month later, a postoperative X-ray was performed (Figure 5).

## Discussion

Non-neoplastic ABCs are expansile bone lesions that possess connective tissue septa, which contain bone trabeculation and osteoclastic giant cells [7]. Roentgenographic and CT scans show an osteolytic lesion that causes the cortical bone around it to grow and shrink. There is frequently a mass of soft tissue. On MR images, ABCs often appear lobulated and multiseptated with fluid-fluid levels and blood degradation products. Simple bone cysts are frequent, benign, fluid-filled cystic lesions that typically develop in the metaphysis of long bones. They induce only minor bone growth. Most often, they affect children and teenagers [8]. Simple bone cysts are a less common differential diagnosis that is considered after the aforementioned diagnostic possibilities have been ruled out. The straightforward bone cyst diagnosis was supported by histological data. Simple bone cyst cases were treated by surgical curettage and bone graft without recurrence, according to Ogata et al. [9]. Some authors have hypothesized a posttraumatic or posthemorrhagic etiology, which could explain the vertebral position, particularly in older people, as the source of solitary bone cysts [3]. The epiphyseal plate has been linked to tumor formation in children; however, in this case, the cyst's peculiar placement suggests that this etiology cannot be the cause. The optimal treatment strategy is still up for debate. Surgery is not typically the preferred course of treatment for young children due to their high likelihood of recurrence, but it may be considered if they present in uncommon anatomical sites such as the spine where recurrence rates are lower. The upper cervical spine was affected in the instance described, and the thinning of the cortex made the bone more prone to fractures that could cause instability or migration of bone fragments into the spinal canal, leading to significant neurological impairment. The surgical method used here was effective.

Children with ABCs in the spine should have their future growth, fusion levels, radiotherapy, and any potential radiation-induced sarcoma, deformity, or postlaminectomy kyphosis taken into account when managing their condition [9]. Back discomfort that gradually worsens is the most typical sign of ABC of the spine, according to research by Lim et al. [10]. Additional symptoms include a palpable spinal tumor, kyphosis or scoliosis, and neurological impairments. In 2011, Novais EN et al. published the largest pediatric case series, which included a retrospective analysis of seven children diagnosed with ABC of the cervical spine at a mean age of 11.9 years [11]. Four of them experienced neurological symptoms such as upper extremity paresthesia and paralysis, and all of them reported signs of neck pain. One case involved the posterior element, C2 vertebral body, and C3 laminae with a significant soft tissue component that caused cord compression in an eight-year-old female patient. Eight months after the last surgical procedure, the patient was under CT control, and the system was successfully stabilized. With the complete removal of the tumor, the reduced chance of relapse, and minimal morbidity, the objective was thus achieved.

## Conclusions

Cervical spine ABCs in children are uncommon. The diagnosis of a cervical spine ABC is not supported by any particular clinical symptoms. Additionally, there is still a need for further scientific and clinical research as well as evidence-based guidelines for ABC treatment. An excellent chance of recovery is linked to the complete excision of ABCs.
